# Exploring the link between emotional states and coding task quality: a pilot study

**DOI:** 10.3389/fnhum.2025.1646242

**Published:** 2025-11-25

**Authors:** Aquib Reshad, Valentina Nino, Maria Valero, Adriane Randolph, Yang Shi

**Affiliations:** 1 Department of Industrial and Systems Engineering, Kennesaw State University, Marietta, GA, United States; 2 Department of Information Technology, Kennesaw State University, Marietta, GA, United States; 3 Department of Information Systems and Security, Kennesaw State University, Marietta, GA, United States; 4 Department of Computer Science, Utah State University, Price, UT, United States

**Keywords:** EEG, emotions, programing quality, Frontal Asymmetry Index, programmers

## Abstract

Emotions play a crucial role in shaping cognitive performance, yet their influence on programing remains understudied. This pilot study investigates the relationship between emotional states and coding task quality. Ten participants completed a programing task while their brain activity was recorded using electroencephalography (EEG), with frontal alpha asymmetry (FAI) applied as a neural marker of emotional valence. Emotional self-reports were collected using the Scale of Positive and Negative Experience (SPANE), and coding quality was evaluated through a structured rubric. Preliminary findings indicate a potential association between FAI and coding performance, whereas self-reported affect showed weaker or inconsistent patterns. Given the small sample size (*n* = 10), these results should be interpreted as exploratory. Nevertheless, the study demonstrates the feasibility of integrating EEG-based emotional measures into software engineering research and lays the groundwork for larger-scale investigations into how emotions influence coding task quality.

## 1 Introduction

Programing is a highly abstract field that underpins the development of robust, efficient applications, enhanced software systems, and a more secure digital environment. Like any other profession, a developer’s performance is influenced by their emotion and wellbeing, and these factors can significantly impact the quality of their work. Prior studies have shown that both positive and negative emotions can affect the quality of code developers produce (Dahlberg, 2020; “Positive Emotion in Sport Performance: Current Status and Future Directions: International Review of Sport and Exercise Psychology: Vol 4, No 1,” McCarthy, 2011; Graziotin et al., 2018; Girardi et al., 2022), though relatively little research has specifically examined the emotional states of developers (Gregory, n.d.) and their direct effects on coding outcomes. This study aims to explore the relationship between programmers’ emotional states and the quality of code they produce, using electroencephalographic (EEG) signals to analyze emotional responses (Li and Lu, 2009). By gaining insights into these relationships, this research could inform strategies for improving team dynamics and workplace conditions (“Decoding Programmers: How Emotions Can Change Code|InformationWeek,” n.d.), ultimately enhancing coding performance and software quality.

Existing research highlights the interplay between programmers’ emotions and their performance, employing diverse methodologies and tools to investigate this relationship. Girardi et al. (2020) conducted an empirical study with 35 participants to explore the connection between emotion and progress, triggers for emotions, and strategies to address negative states, identifying non-invasive biometric sensors suitable for emotion recognition during programing tasks. Similarly, various physiological methods were explored by another project (Wrobel, 2018), including video analysis, skin conductance, heart rate, respiration, and facial electromyography (fEMG), to assess their applicability during programing. In Cagnoni et al. (2020), the authors analyzed emotional trends expressed by developers in 2018 Stack Overflow posts across 26 programing languages, revealing significant language-specific patterns. In other research (Lishinski et al., 2017), the authors piloted a basic emotional reactions survey among undergraduate programing students, offering insights into how emotions influence learning in programing education. Furthermore, empirical evidence was provided linking emotions to perceived productivity in the workplace (Girardi et al., 2022). Additionally, Gachechiladze et al. (2017) developed a classifier to automatically detect anger direction by mining comments in Apache issue reports. While these studies underscore the importance of emotions in programing contexts, the use of EEG devices to investigate programmers’ emotional states and their effect on coding quality remains relatively unexplored.

In this research, frontal asymmetry was used to differentiate between the negative and positive emotional states of the participants. Frontal asymmetry, particularly in the alpha band, has emerged as a significant indicator of emotional states and traits, as established by extensive research. Originally discovered by Richard Davidson and colleagues (Hagemann et al., 2002) and linked to personality, subsequent studies have demonstrated its broader relevance in emotion processing and mental health (Gollan et al., 2014; Mikolajczak et al., 2010). We only focused on the alpha band for this study because frontal alpha asymmetry has been extensively validated in prior literature as a neural correlate of emotional valence, approach—withdrawal motivation, and stress regulation. While other frequency bands (theta, beta) may also provide relevant information, focusing on alpha allowed for a more targeted and theoretically grounded analysis. Alpha activity in EEG signals inversely reflects cortical activity—lower alpha power indicates higher cortical activity, while higher alpha power corresponds to reduced activity (Bazanova and Vernon, 2014). This dynamic makes Frontal Asymmetry Index (FAI) a powerful tool for exploring brain activity associated with emotional regulation and perception. For instance, differences in frontal alpha asymmetry have been linked to emotional responses, including traits like anxiety and mood disorders, and manipulated asymmetry has shown impacts on emotion regulation capabilities (Papousek et al., 2011). The ability of FAI to provide non-invasive insights into the neural underpinnings of emotions highlights its importance in both research and applied settings, including mental health interventions and understanding workplace behaviors. Recognizing this gap of usages of EEG, the current study measures emotions using Frontal Asymmetry Index through EEG and aims to establish a relationship between emotional states and coding performance, offering a novel perspective in understanding and enhancing developer productivity.

## 2 Materials and methods

### 2.1 Participants

Ten student participants (8 males and 2 females) from various university departments, with an average age of 28.33 ± 2.68 years and at least 18 years old, were recruited for the study. All participants had to complete a pre-study questionnaire for an initial pre-screening process to ensure that they had a foundational knowledge of Python. This initial pre-screening process was completed via email in which their eligibility was established by completing basic programing questions. The procedure served to screen out those who may have lacked the essential programing skills, ensuring that all participants were appropriate for the study’s coding challenge. Individuals were excluded if they had a history of depression or other mental health conditions or showed any symptoms of COVID-19. Each participant attended one in-person lab session lasting up to 1.5 h. Since this study needed to collect data from human subjects, consent was obtained for each participant (IRB Number: IRB-FY23-166) before the acquisition of data.

### 2.2 Data acquisition

Upon arrival at the lab, participants were given a Consent Form to read and sign before proceeding. Then, they took a demographic and self-reported survey about their background, health habits and conditions such as caffeine usage, emotional state (Rested, Alertness, Stress, Anxiety), and responses to the SPANE (Scale of Positive and Negative Experience) questionnaire (“SPANE—Ed Diener, Subjective Wellbeing,” Diener, 2024). They then described the neuroheadset and its purpose, before having the headset fitted for EEG data collecting. The EEG recording system was the BioSemi ActiveTwo bio-amplifier system (“Biosemi EEG ECG EMG BSPM NEURO Amplifier Electrodes,” Biosemi, 2025). The ActiveTwo system consists of two components: a headbox that links to a set of electrodes put on the scalp to measure EEG signals and a bio-amplifier unit that magnifies and digitizes these signals. A 1-min baseline EEG recording was taken while the individual stared at a blank screen. Once this was completed, individuals started an hour-long coding assignment in which their brain signals were recorded. The assigned task was a frequently used version of the rainfall problem (“Do We Know How Difficult the Rainfall Problem Is?|Proceedings of the 15th Koli Calling Conference on Computing Education Research,” Seppälä et al., 2015). Participants were required to write a Python script that calculated the average of a series of non-negative integers entered by the user. The program would continuously accept inputs and, upon encountering the number 99,999, output the average of all preceding values. Participants could cease participating in the research at any moment, and after the activity was completed or the time limit had expired, the neuroheadset was removed to end the session.

### 2.3 Measures

#### 2.3.1 Coding quality

To evaluate the students’ scripts, each was assessed based on five criteria, with each fulfilled criterion awarded one point, for a maximum score of five. The rubric used was as follows:

- Loop structure: Whether the student successfully implemented a “for” or “while” loop to iterate through the inputs.
- Average calculation: Whether the algorithm for calculating the average of numbers was correct.
- Input handling: Whether the student effectively managed the input format and structure.
- Exit and output: Whether the program correctly exited upon encountering 99,999 and printed the average.
- Edge case handling: Whether the code handles special cases, such as an immediate 99,999 input.

Each criterion fulfilled earned one point, providing a clear, quantitative measure of the student’s proficiency in achieving the coding task requirements.

#### 2.3.2 The Frontal Asymmetry Index

This is a metric derived from EEG signals that reflects the balance of brain activity between the left and right frontal regions, particularly in the alpha frequency band (8–12 Hz) (Zsigo et al., 2024). It is often used in psychological and neuroscience studies to assess emotional and cognitive states, such as motivation, mood, and approach/avoidance behaviors. Frontal Asymmetry Index is based on the concept of hemispheric asymmetry, which posits that the left and right hemispheres of the brain are specialized for different types of emotional processing. Specifically:

– Left frontal activity is generally associated with approach-related behaviors, positive emotions, and motivation (e.g., joy, excitement).
– Right frontal activity is typically linked to avoidance behaviors, negative emotions, and withdrawal (e.g., fear, sadness).

#### 2.3.3 Scale of positive and negative experience

The SPANE is a psychological assessment test that measures an individual’s emotional experiences, including both positive and negative emotions (Jovanović, 2015). The SPANE, developed by Ed Diener and colleagues, is a brief yet comprehensive technique to understand how individuals feel across time, and it is often used in wellbeing research. The SPANE is frequently used in conjunction with other wellbeing indicators, such as life satisfaction scores, to provide a more nuanced picture of a person’s emotional and psychological health. It is especially prized for its simplicity and capacity to express a wide range of emotions, including generic feelings (e.g., “pleasant,” “unpleasant”) as well as more specific emotions such as “joyful” or “angry.”

#### 2.3.4 Self-reported emotional state

In addition, the participants had to complete a questionnaire regarding how rested they felt, their level of alertness, stress, and anxiety at that moment of time. They assigned a number on a scale of 1–5, with five representing the most intense.

### 2.4 Data processing

The FAI was calculated from EEG data through a series of processes, beginning with data cleaning and filtering. After loading the raw EEG data, a bandpass filter was used to maintain the frequency components between 1 and 40 Hz. This filtering stage removed both low-frequency noise (such as gradual drifts) and high-frequency artifacts (such as muscle noise), preserving only brain activity within this range. This is critical for focusing on relevant brain signals, notably the alpha band (8–12 Hz), which is associated with cognitive and emotional functions. Independent Component Analysis (ICA) was also applied to detect and remove components related to eye blinks, muscle movements, and line noise.

Once filtered, the data was divided into time segments for analysis. The first minute of data was taken as the starting segment, while the remainder was separated into 5-min sections. If any data remained after these 5-min segments, it was considered the final section. This segmentation was critical for assessing variations in brain activity over time, providing for a more in-depth knowledge of how the FAI varied throughout the recording.

Following data splitting, Welch’s approach (Bandpower of an EEG Signal, 2018) was used to calculate alpha band power (8–12 Hz) for each relevant channel. This method determined the power spectral density of the signal, allowing the average power in the alpha frequency range to be calculated. Alpha Band Power is particularly interesting in the frontal channels (F3 and F4), which are important regions for emotional and cognitive functions.

Finally, the FAI was determined by taking the natural log of F4’s alpha band power (right frontal) and subtracting F3’s alpha band power (left frontal). This measures the relative difference in activity between the left and right frontal lobes.

SPANE consists of 12 items, with six items assessing positive feelings (e.g., “happy,” “joyful,” “content”) and six items assessing negative feelings (e.g., “sad,” “afraid,” “angry”). Respondents were asked to reflect on their emotional experiences over the past 4 weeks and rated each item on a scale from 1 (Very Rarely or Never) to 5 (Very Often or Always).

The scale generates two sub-scores:

- SPANE-P: The sum of the scores for positive feelings.
- SPANE-N: The sum of the scores for negative feelings.

In addition to these sub-scores, a balance score (SPANE-B) can be obtained by subtracting the negative score (SPANE-N) from the positive score (SPANE-P). This balancing score reflects the total emotional experience, with higher positive scores indicating more pleasant emotions and lower or negative values indicating a predominance of negative feelings (Diener et al., 2009).

Finally, emotional states (Rested, Alertness, Stress, Anxiety) were measured using the answers provided by participants on the questionnaire.

To investigate potential relationships, we initially plotted graphs with coding quality as the independent variable and various dependent variables, including Rested, Alertness, Stress, Anxiety, FAI, and SPANE values. This visual examination allowed us to observe any emerging patterns or correlations between coding quality and each variable. When notable patterns appeared, we calculated Pearson correlation coefficients to assess the strength and direction of these relationships quantitatively (Sedgwick, 2012).

## 3 Results

The average task completion time was 2316 ± 1141.38 s, and the average coding score was 3.2, with four of the 10 participants achieving the highest score of 5 and one scoring 0. Regarding pre-task conditions, one participant smoked, two were hungry, and one consumed alcohol 10 h before the task. The time since the participants’ last major meal averaged 6.45 ± 6.17 h, ranging from 0.45 to 15.5 h. Now, below are the results from the assessments conducted.

### 3.1 SPANE score

**FIGURE 1:**
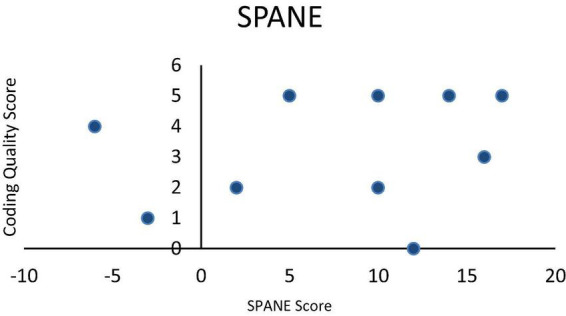
SPANE score vs. coding quality score.

The SPANE-B scores, which measured participants’ balance of positive and negative emotions, had a mean of 7.7 ± 7.93, ranging from a minimum of −6 to a maximum of 17. Both the positive and negative feelings subscales recorded an average score of 21.7. Among the participants, two reported negative SPANE-B scores, indicating a predominance of negative emotions, while the remaining participants had positive scores. Notably, four individuals with positive scores had values of 10 or less, reflecting moderate emotional states despite an overall positive balance. Higher SPANE-B scores, indicating more positive emotions, are generally associated with higher coding scores, but there are exceptions with lower coding scores at both positive and negative SPANE-B values. The results show no consistent trend is clearly observed in Figure 1.

### 3.2 Emotional state score

**FIGURE 2:**
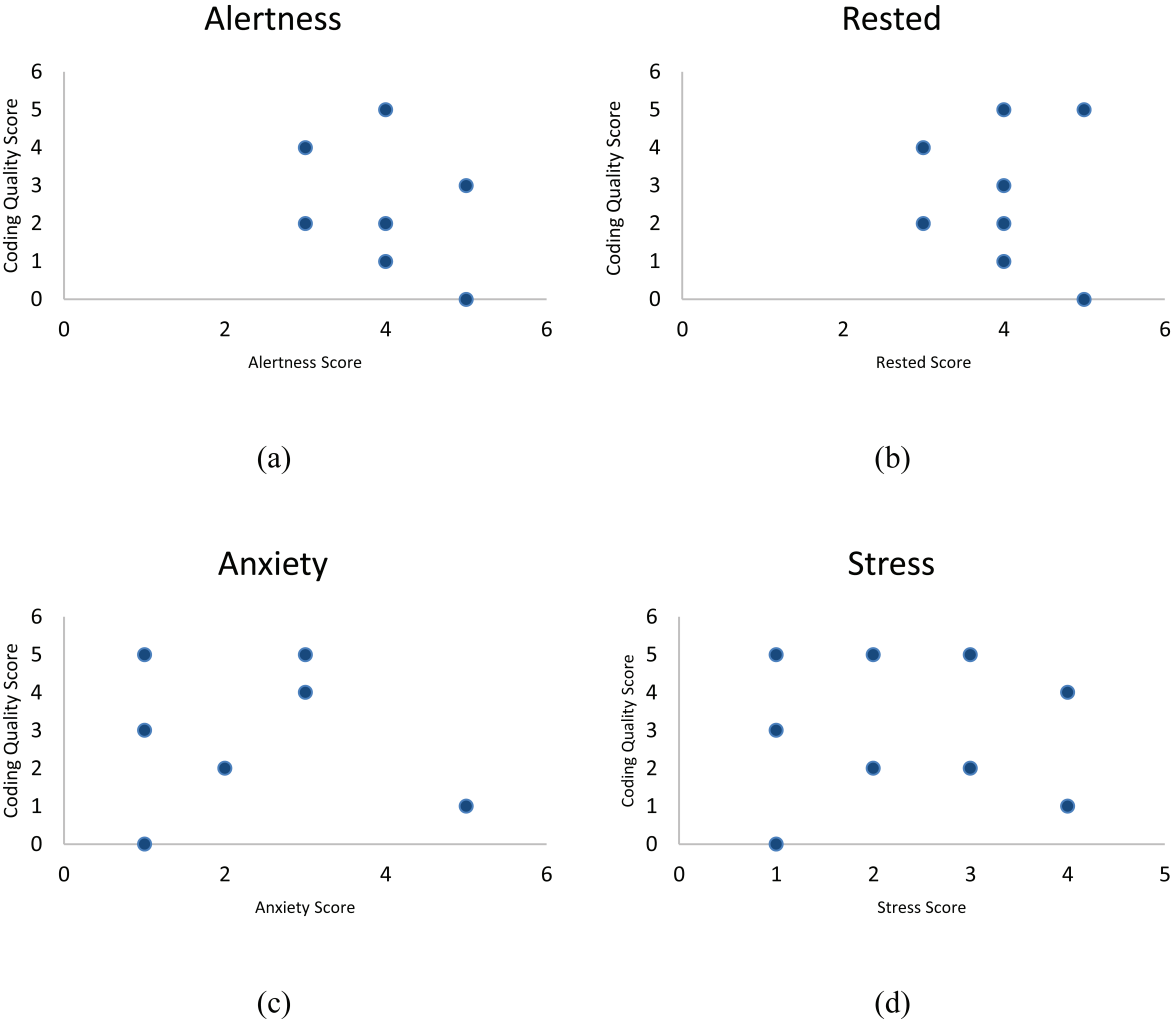
**(a)** Alertness vs. coding quality score; **(b)** rested vs. coding quality score; **(c)** anxiety vs. coding quality score; **(d)** stress vs. coding quality score.

Participants rated their emotional states across four dimensions: restedness, alertness, stress, and anxiety. On average, participants reported feeling well-rested and alert, with both categories receiving an average score of 4. Two participants rated themselves at the highest level (5) for both restedness and alertness. Stress levels were moderate, with an average score of 2.4. No one rated the highest stress level of 5, but two participants scored a 4, while three reported the lowest stress level of 1. Anxiety levels were similarly moderate, with an average score of 2.2. One participant reported the highest anxiety level of 5, while four participants rated the lowest level of 1. These findings suggest that most participants were generally rested and alert, experiencing moderate levels of stress and anxiety. The alertness values ranged from approximately 3 to 5, and coding scores span from 0 to 5. In Figure 2a, there seems to be no clear trend between the two variables, as the coding scores are spread across different alertness levels. Similarly in Figure 2b, the coding scores are distributed across different levels of being rested, with no obvious trend or correlation between the two variables. There appears to be a higher concentration of coding scores at lower anxiety levels (around 1 and 3), and as anxiety increases, the coding scores become more variable, with some lower coding scores at higher anxiety levels as per Figure 2c. However, no clear relationship between anxiety and coding scores is apparent. Lastly, Figure 2d shows no clear pattern between coding scores and stress levels, with both high and low coding scores appearing at various stress levels.

### 3.3 Frontal Asymmetry Index score

**FIGURE 3:**
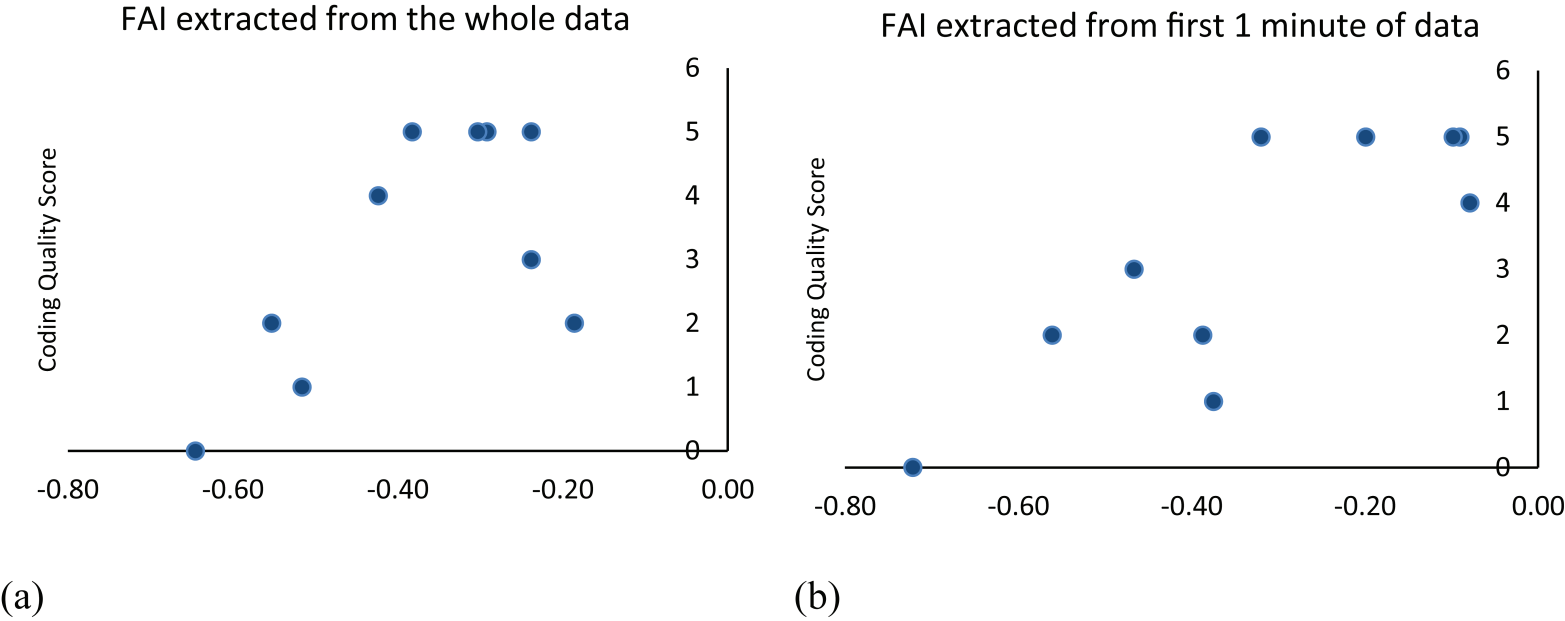
**(a)** Frontal Asymmetry Index extracted from the whole data vs. score graph vs. coding quality score; **(b)** Frontal Asymmetry Index extracted from the first 1 min of data vs. coding quality score.

As per Figure 3a, the variability in FAI (extracted over whole data collection process) across different coding scores indicates a complex interplay, with lower FAI values (suggesting stronger right activation) found in both high and low coding scores, implying that the relationship between frontal asymmetry and coding performance is not straightforward. On the other hand, Figure 3b illustrates the relationship between “Coding Score” and the FAI for the first minute of data collection. Coding scores ranged from 0 to 5, with predominantly negative FAI values indicating greater activation in the right frontal region (F4) compared to the left (F3). A Pearson correlation coefficient of approximately 0.83 suggests a strong positive correlation, meaning higher coding performance was linked to less negative FAI, potentially reflecting increased left frontal activity.

**FIGURE 4:**
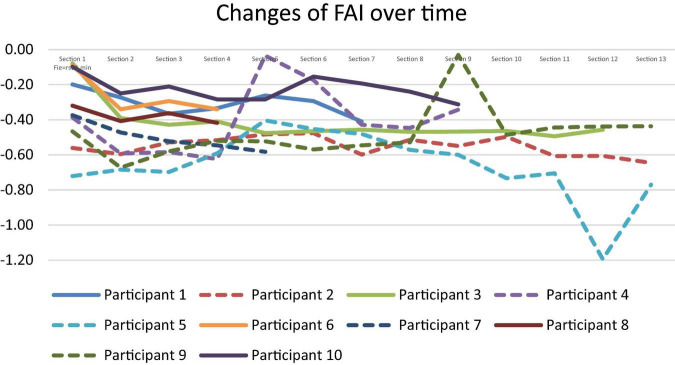
Changes of FAI for 10 participants (dotted line for scores 3 or less).

In examining the graph of FAI changes over time in Figure 4, slight differences between participants with higher coding quality scores (solid lines) and those with lower scores (dotted lines) were observed. Participants with lower scores generally showed more fluctuating and often more negative FAI values across the sections, indicating potentially higher right-frontal dominance, which can be associated with increased stress or frustration. Conversely, participants with higher scores (solid lines) appeared to have relatively stable FAI values, often remaining closer to zero or showing less drastic fluctuations, which might suggest a more balanced or less negative emotional state during the task. This distinction between the stable trends for higher performers and the fluctuating pattern for lower performers highlights a potential relationship between FAI stability and coding performance quality. The correlations across self-reported emotional measures were weak and inconsistent, and only the pre-task FAI showed a strong positive association with coding score. In future studies, more rigorous statistical modeling (e.g., mixed-effects regression, multiple-comparison correction) will be applied.

### 3.4 Brain mapping with sLORETA

**FIGURE 5:**
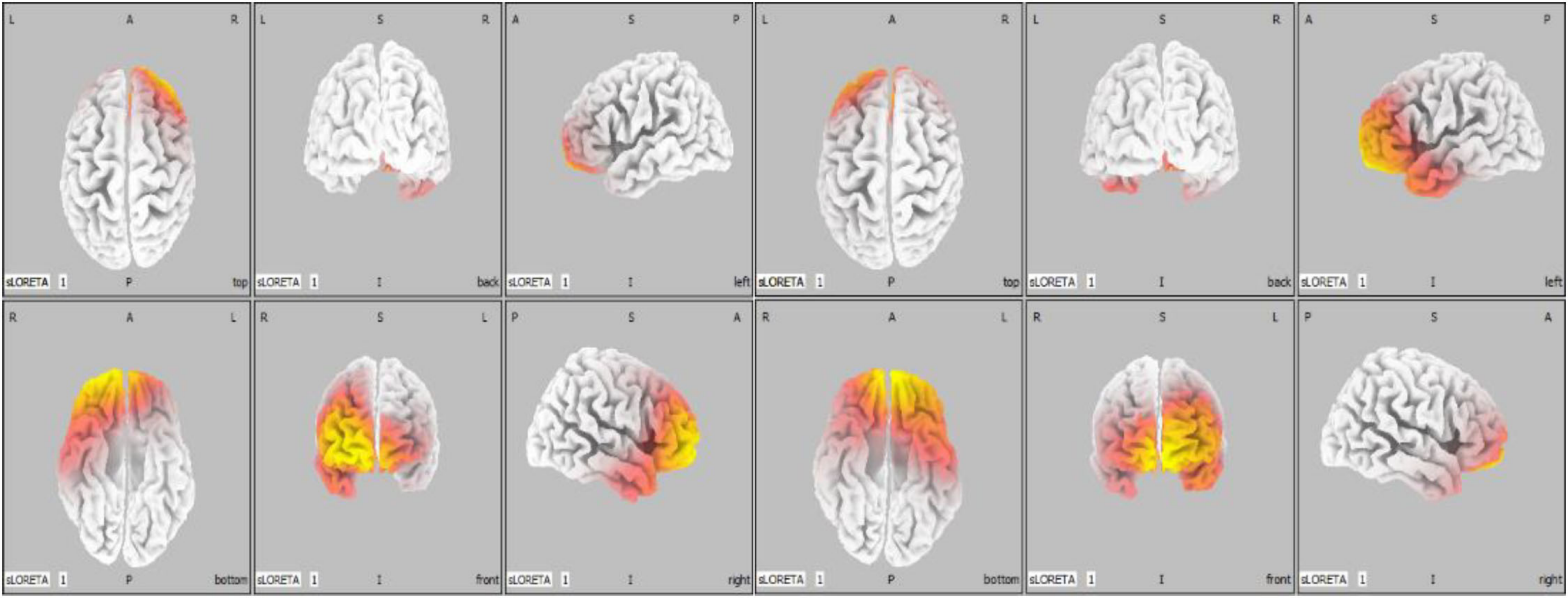
Brain mapping result for two different participants (left: right side is more active, right: left side is more active).

Standardized Low-Resolution Brain Electromagnetic Tomography (sLORETA) provides a six-view brain mapping representation that helps visualize neural activity across different brain regions (Tejay and Mohammed, 2023; Pascual-Marqui, 2002). The six-view representation that can be seen in Figure 5 helped to identify whether increased activity was localized in the left or right hemisphere.

Based on the sLORETA results in Table 1, there appeared to be no clear trend in hemispheric activation concerning performance scores. Among the participants who scored 4 or 5, three exhibited the right hemisphere activation, while three showed left hemisphere activation, indicating no consistent lateralization pattern for higher performance.

**TABLE 1:** sLORETA brain mapping result with score.

Participants number	Score	Area that is more active (left/right)
Participant 1	5	L
Participant 2	2	R
Participant 3	4	R
Participant 4	2	L
Participant 5	0	L
Participant 6	5	R
Participant 7	1	L
Participant 8	5	R
Participant 9	3	R
Participant 10	5	L

Conversely, for participants with lower scores (0–3), two displayed left hemisphere activation, and two showed right hemisphere activation. This mixed activation pattern suggests that while frontal asymmetry was present, it did not consistently correlate with performance levels in a straightforward manner.

## 4 Discussion

The relationship between emotional states and coding performance, as reflected in SPANE-B scores, reveals intriguing yet nuanced patterns. Higher SPANE-B scores, indicative of more positive emotional states, were generally associated with higher coding quality. This finding aligns with the notion that positive emotions can enhance cognitive performance and problem-solving abilities by fostering a more engaged and motivated mindset (Chuang, 2007). However, exceptions were noted, with some participants producing lower-quality code despite reporting both positive and negative SPANE-B values. These inconsistencies suggest that while emotional positivity may generally support better performance, other factors, such as task complexity, individual differences in skill level, or external distractions, may moderate this relationship (Dieste et al., 2017).

The lack of clear trends for alertness, state of rest, anxiety, and stress scores in relation to coding quality is particularly noteworthy. This suggests that the influence of these states on performance may vary greatly among individuals or require more precise measurement tools to capture their true impact. For example, self-reported measures might not fully reflect participants’ actual mental states during the task, underscoring the need for objective, physiological, and neurological measures in future studies. Additionally, this finding highlights the potential complexity of emotional and cognitive interactions in programing tasks, where factors like skill level, experience, or even task familiarity might play significant roles that were not directly assessed in this study. Moreover, the findings, based on sLORETA brain mapping, indicate that brain activity patterns during the task are not strictly oriented laterally though the FAI provided more consistent and actionable insights. A strong positive correlation was observed between coding performance and less negative FAI values that were extracted over the first 1 min before starting the task, reflecting increased left frontal activity. This value, extracted prior to the task, reflects the participant’s initial neural state. The overall FAI also showed some trend with the coding performance though was not as strong as the first 1-min data. This finding aligns with existing literature (Gregory, n.d.) that associates left frontal dominance with approach-oriented behaviors, cognitive engagement, and task focus. Furthermore, the observed overall changes in FAI indicate that participants with lower scores not only exhibited lower initial FAI values but also experienced more pronounced fluctuations in FAI throughout the task duration. The observed relationship suggests that FAI could serve as a neural marker for assessing cognitive workload and task-related engagement, offering potential applications in real-time monitoring of programing performance. For instance, workplaces could implement neurofeedback systems to track FAI and provide interventions such as tailored breaks or stress management programs to maintain productivity. Similarly, educational tools leveraging FAI could help personalize training programs, ensuring that learners remain engaged and focused on their tasks.

**FIGURE 6:**
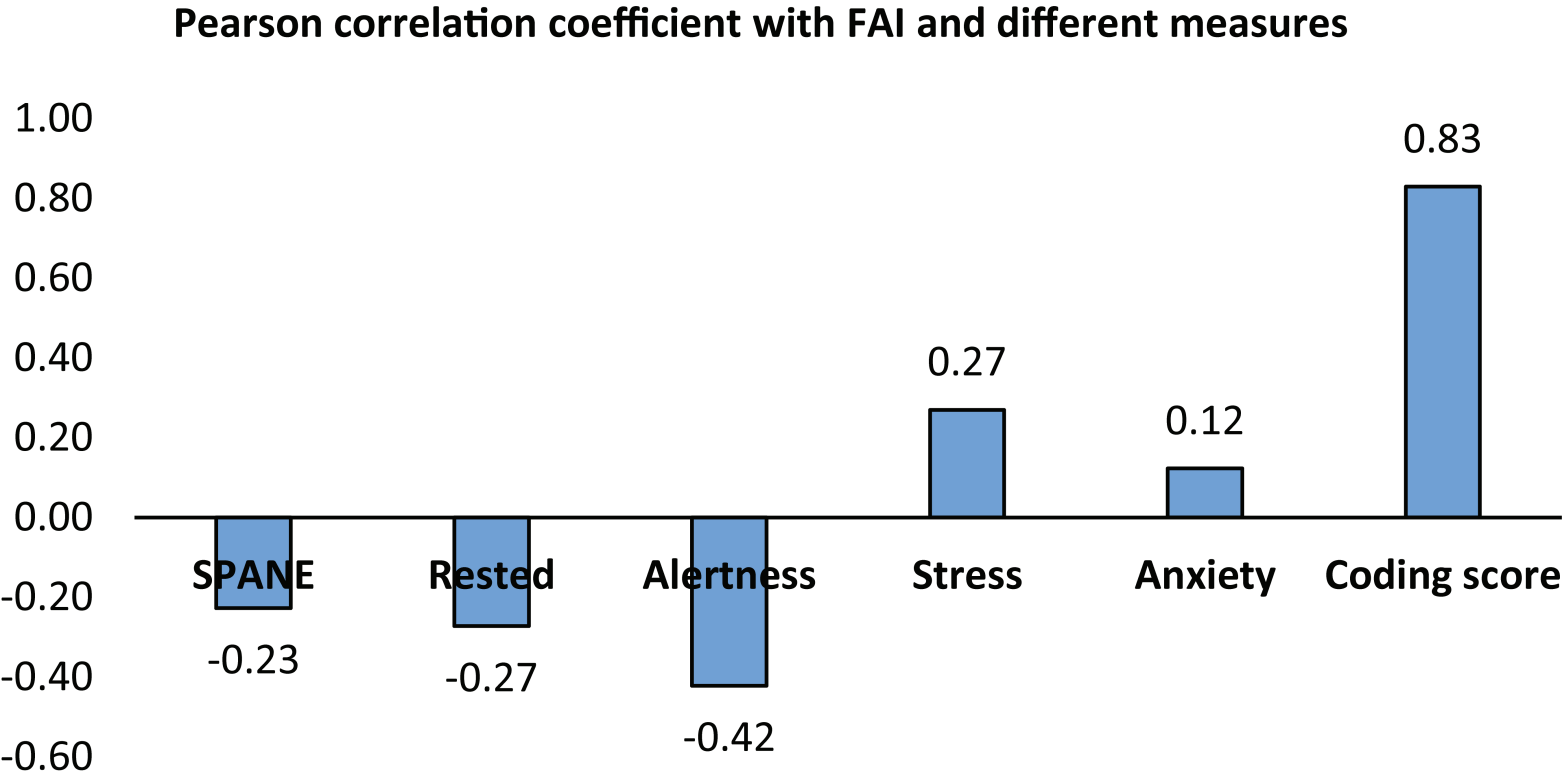
Correlation between FAI and the different measures.

The consolidated results in Figure 6 shows the relationship between the FAI, extracted 1 min before the coding task, and various measures. Frontal Asymmetry Index exhibits a weak negative correlation with SPANE (−0.23), rested (−0.27), and alertness (−0.42), indicating that higher left frontal activity (reflected by less negative FAI) is associated with lower emotional positivity, reduced feelings of rest, and decreased alertness. Since higher left frontal activity is related to greater approach motivation, the correlation with SPANE score shows a weak negative relationship with positive emotions and self-reported emotional positivity. Again, the self-reported Rested and Alertness score also shows an opposing relationship owing to the fact that positive emotions are seen as per the EEG recordings, but the self-reported questionnaire shows the participant is tired and less alert. Conversely, FAI has a weak positive correlation with stress (0.27) and anxiety (0.12), suggesting that higher FAI values may relate to slightly higher stress and anxiety levels. Notably, FAI demonstrates a strong positive correlation with coding scores (0.83), highlighting that increased left frontal activity indicating a positive emotion before the task is associated with better coding performance. This significant relationship underscores the potential of FAI as an indicator of cognitive performance and its role in emotional and mental states during tasks (Chai et al., 2014; Bayazi et al., 2023).

Despite these promising insights, the study has several limitations. First, the sample size was small (*n* = 10), limiting the generalizability of the findings although other studies indicate that little may change with increased sample size [13]. Second, the gender distribution was unbalanced, with eight male and only two female participants, which may have influenced the results and reduced the representativeness of the study. Third, the reliance on self-reported questionnaires introduces potential biases, as participants may have misunderstood certain questions or chosen not to disclose certain information, affecting the accuracy of the collected data. Fourth, the absence of real-world task conditions might limit the ecological validity of the findings, as coding tasks in controlled settings may not fully capture the complexities of workplace environments. Future studies should address these limitations by recruiting larger, more diverse participant groups and incorporating more objective measures of emotional states to validate and expand upon these findings.

This study’s findings underscore the multifaceted relationship between emotional states and programing performance, shedding light on how neural and emotional markers like FAI can influence task outcomes. But it is important to emphasize that the present findings are preliminary in nature, given the limited sample size and exploratory design of the study. Rather than providing definitive evidence, the results serve as an initial step that allowed the research team to generate hypotheses regarding the potential influence of emotional states on coding quality. These hypotheses can guide future studies with larger and more diverse samples, more controlled experimental conditions, and refined analytical methods to validate and expand upon the trends observed here. By building on this research, we can move closer to creating adaptive, emotionally aware environments that support developers’ wellbeing and optimize their productivity. Such advancements could lead to practical applications in workplace management and educational settings, paving the way for enhanced performance and mental health outcomes in software development and other cognitively demanding fields. The practical implications of this relationship are significant, particularly in the context of workplace productivity and individual performance optimization. Monitoring FAI could provide real-time feedback on a developer’s cognitive and emotional state, offering opportunities to identify periods of stress, disengagement, or fatigue that may impact coding quality. For example, organizations could integrate neurofeedback tools to assess and support programmers during high-stakes projects, allowing for timely interventions such as breaks, task adjustments, or stress management strategies.

Additionally, the relationship between FAI and coding performance has potential applications in education and training. By understanding how frontal asymmetry reflects task engagement and performance, educators could tailor programing assignments and learning environments to better align with students’ cognitive and emotional states. Furthermore, these insights could be extended to team dynamics, where FAI data might be used to optimize collaboration by identifying individuals best suited for specific tasks based on their cognitive-emotional profiles. This study underscores the value of incorporating neurophysiological measures into the evaluation and improvement of programing outcomes, though further research with larger and more diverse samples is needed to fully explore these applications.

## 5 Conclusion

The findings suggest that negative emotional states (lower FAI) may be linked to the production of lower-quality code by programmers, a result that aligns with earlier research findings (Gregory, Emotional Code, 2021; Haehl et al., 2020). While this observation is intriguing, the small sample size used in this study (*n* = 10) limits the robustness and generalizability of the conclusions. On the other hand, some other studies showed the opposite of the result found here (“Decoding Programmers: How Emotions Can Change Code|InformationWeek,” InformationWeek, 2024; Forgas, 2007). This limitation underscores the need for further studies involving a larger and more diverse participant pool to ensure statistical power and allow robust modeling in order to better evaluate and clarify the nature of the relationship between emotional states and coding performance. With the small cohort size, we were also unable to conduct subgroup contrasts, but this is an important direction. Future studies will implement clustering (e.g., high vs. low performers, positive vs. negative emotional states) and canonical correlation analyses to reveal group-level effects. Moreover, the current sLORETA results did not reveal consistent lateralization. In future studies, we will limit claims from the sLORETA results, frame them as exploratory, and emphasize that meaningful group-level contrasts require a larger dataset. While FAI was our primary physiological marker, future iterations of the study may include additional measures such as heart rate variability (HRV) or galvanic skin response (GSR) to further validate emotional states. Furthermore, coding quality was not assessed in a time-segmented manner; rather, the final code was evaluated holistically based on predefined criteria. While this approach allowed for a consistent and objective assessment across participants, it limited the ability to examine temporal trends in code quality throughout the task. For future research, implementing time-resolved evaluations of coding performance could provide valuable insights into how cognitive workload and fatigue influence programing behaviors over the course of the session. Lastly, factors such as caffeine, sleep, hunger, and alcohol were not adequately controlled, and that this is a limitation. These factors will be more strictly monitored in future experiments. Moreover, the lack of a neutral or control task is a valid critique. In the next phase of research, we plan to include baseline tasks (e.g., arithmetic, reading comprehension) to separate emotional influences from general cognitive. A more extensive investigation could provide critical insights into how emotions influence programing outcomes. Such insights would not only deepen our understanding of the complex dynamics between emotional states and technical performance but could also inform strategies for fostering productive and supportive environments for developers. One of the long-term goal is to leverage these findings toward developing real-time monitoring and feedback systems. Such systems could provide adaptive interventions (e.g., breaks, task suggestions, or supportive feedback) for programmers experiencing elevated stress or negative effects, ultimately enhancing productivity and wellbeing This would have implications for optimizing both individual performance and team productivity in programing-focused occupational settings.

## Data Availability

The raw data supporting the conclusions of this article will be made available by the authors, without undue reservation.
